# Internalising and Externalising Symptoms and Their Association with the Family Environment in Young Children with Williams Syndrome: A Longitudinal Study

**DOI:** 10.3390/children10101717

**Published:** 2023-10-23

**Authors:** Tamara Ilic, Melanie A. Porter, Jessica L. Reeve

**Affiliations:** School of Psychological Sciences, Macquarie University, Balaclava Road, Marsfield, Sydney, NSW 2109, Australia; tamara.ilic@students.mq.edu.au (T.I.); jessica.reeve@mq.edu.au (J.L.R.)

**Keywords:** williams syndrome, WS, psychopathology, family environment, child behaviour checklist, CBCL, internalising, externalising

## Abstract

Williams Syndrome (WS) involves high rates of psychopathology across the lifespan. However, little is known about the early, longitudinal trajectory of internalising/externalising symptoms or the association between these and the family environment in WS. WS (*n* = 16; aged 2 years, 2 months to 9 years, 5 months) and typically developing or TD (*n* = 46; aged 2 years, 2 months to 11 years, 1 month) children were assessed on two occasions over 2.5 years utilising parent report questionnaires—the Child Behaviour Checklist and the Family Environment Scale. No statistically significant changes were found in CBCL/psychopathology profiles across timepoints, on average, for either WS or TD children. However, reliable change scores showed WS children had considerable variability in CBCL scores over time. Cross-sectionally, the WS group showed higher scores (reflecting more psychopathology) compared to TD controls at both time points across most CBCL subscales, with elevated overall psychopathology problems identified in 56–68% of WS children (versus 8% in TD controls). Psychopathology was not associated with sex, chronological age, or cognitive ability in WS. Conflict in the family environment was positively associated with higher Attention Problems at Time 1 in the WS group, whilst the TD group showed associations between family conflict and total psychopathology problems at both time points and between family cohesion and total psychopathology problems at Time 2. Family environment did not differ between groups, except for lower engagement in intellectual and cultural activities in WS. Findings highlight variable Internalising and Externalising Problems in young WS children over time, with greater biological than environmental contributions to psychopathology in WS.

## 1. Introduction

Williams Syndrome (WS) is a rare genetic condition arising from a microdeletion on chromosome 7 at the location 7q11.23 [1]. WS occurs in 1 per 7500 to 1 per 20,000 individuals, affecting males and females equally [1] It is a multi-system condition, affecting endocrine, gastrointestinal, musculoskeletal, cardiovascular, and central nervous systems [1]. Neurocognitively, WS is associated with intellectual disability, typically in the mild-to-moderate range, and a profile of cognitive strengths and weaknesses [1]. High prevalence rates of comorbid neurodevelopmental conditions (i.e., Attention-Deficit Hyperactivity Disorder (ADHD; 33–80%) and Autism Spectrum Disorder [12–20%]) and psychiatric disorders (i.e., anxiety disorders [37–65%] and mood disorders [3–25%]) occur in WS individuals, with the prevalence varying somewhat across the lifespan [1].

Families of WS individuals are impacted by the unique challenges associated with supporting a person with a disability and are significantly more likely to experience anxiety and stress compared to the general population [2,3]. Research on typically developing individuals has also shown that family functioning, including family environment characteristics, is associated with psychopathology outcomes [4,5,6]. Despite this, no research has explored the association between psychopathology in WS and the family environment. The current study had two primary aims: (1) to examine the longitudinal trajectory of psychopathological functioning in WS and (2) to investigate the relationship between the family environment and psychopathological symptomology, both cross-sectionally and longitudinally. As a sub-aim, we also investigated the relationship between demographic factors, intellectual ability, and psychopathology. A particular focus was made on the distinction between internalising (i.e., problems with anxiety, depression, and somatisation) and externalising (i.e., behavioural symptoms, such as attention problems, hyperactivity, and aggression), consistent with the extant literature in typically developing children when looking at the relation between the family environment and psychopathology [4,5,6].

### 1.1. Internalising and Externalising Symptoms in WS

Internalising and externalising impairments are commonly reported in the WS population, with the nature, prevalence, and severity seemingly fluctuating over the lifespan [1,7,8]. Generally, studies report high rates of anxiety, depression, obsessions/compulsions, inattention, poor sleep, and reduced social functioning in WS, and these difficulties, particularly Internalising Problems, tend to increase over time [1,7,8,9,10,11]. 

As most WS samples are mixed and comprise adolescents and adults, very little is known about the development of Internalising and Externalising Problems in preschool- to primary-school-age children with WS [1]. Longitudinal research utilising diagnostic interviews (such as the Anxiety Disorders Interview Schedule (ADIS); [12]) and parent report questionnaires (i.e., Developmental Behaviour Checklist (DBC); [13]) have found that the majority (62%) of young WS children present with chronic anxiety disorders [14] and demonstrate elevated and chronic emotional and behavioural disturbance compared to typically developing children over time [15,16]. The majority of cross-sectional research with early childhood WS samples (e.g., [3,17,18,19,20,21,22,23] has utilised the Child Behaviour Checklist (CBCL; [24]), a commonly used assessment of childhood internalising and externalising symptoms which map onto empirically based syndrome scales and scales based on the Diagnostic and Statistical Manual of Mental Disorders, Fifth Edition (DSM-5; [25]), referred to as DSM-oriented scales. Overall, studies utilising the CBCL indicate that, on average, 22 to 33% of preschool- to school-aged WS children present with total psychopathology problems in the borderline to clinically significant range [21,22,23], with significantly higher Total Problems identified in WS children than children with other genetic and neurodevelopmental disorders (e.g., Down Syndrome or Prader-Willi Syndrome) and typically developing controls [20,22]. Elevated Internalising Problems are also reported to occur in 10 to 28% of WS children, with particular difficulties in Anxious/Depressed symptoms (5–26%), Withdrawn/Depressed symptoms (11–15%), and Somatic Complaints (14–28%), as well as DSM-orientated Affective Problems (20–33%) and Anxiety Problems (9–37%) [17,21,22,23]. Elevated Externalising Problems are identified in 11 to 25% of preschool- to school-aged WS children and consist of significant Attention Problems (40–69%), Rule-Breaking Behaviour (9–13%), and Aggressive Behaviour (3–21%), as well as DSM-orientated Attention-Deficit Hyperactivity Problems (17–58%), Oppositional Defiant Problems (6–14%), and Conduct Problems (7–13%) [17,21,22,23]. There is fluctuation in the specific rates of elevated psychopathology symptoms; however, studies generally concur that the main areas of concern in young WS children include problems with attention, anxiety, somatic complaints, and emotion dysregulation, although because most measures are screening tools and not diagnostic measures, and given their young age, it is not clear whether such symptoms warrant clinical diagnoses [3,17,21,22]. Another notable limitation of the existing literature is the inconsistent reporting of results, particularly the tendency to conflate subclinical and clinical scores (rather than considering clinically significant scores separately) and the reporting of incomplete CBCL profiles. The trajectory of Internalising and Externalising Problems in young WS children also warrants further investigation. Whilst the CBCL is the most commonly used assessment tool in research conducted with young WS children, all studies utilising this measure in a young WS sample have been cross-sectional in nature, and early longitudinal CBCL profiles have not been investigated. As such, further research is essential in order to explore CBCL profiles over time during the early development of WS individuals to inform early intervention and management planning of Internalising and Externalising Problems.

### 1.2. Effect of Demographic Variables and Cognitive Ability

Only a few studies have examined sex differences on the CBCL in young WS children, and findings are mixed, with Neo and Tonnsen [22] and Perez-Garcia et al. [23] finding no significant sex differences in CBCL profiles, whilst Klein-Tasman and Lee [21] identified that males were rated as significantly higher in Affective Problems than females; however, this was not the case in any other subscale. The literature examining older childhood, adolescent, and adult WS samples has similarly found inconsistent findings, ranging from no sex differences [9,26] to higher prevalence rates in females compared to males in Externalising Problems, Affective Problems, and Somatic Complaints (e.g., [27]). In early childhood, chronological age has been significantly and positively correlated with higher Internalising and Externalising Problems [20,22,23], whilst in older childhood/adolescent samples, chronological age has been significantly associated with higher Internalising Problems but not Externalising Problems [26].

Whilst some studies have failed to find an association between IQ and CBCL profiles [19,21], others note that this association may vary between the following: (1) culture (e.g., positive associations identified in American and not Spanish cultures; Perez-Garcia et al. [23]; (2) chronological age (e.g., IQ is associated with Externalising Problems in younger WS children and Internalising Problems in WS adolescents [9]; and (3) CBCL subscale (e.g., association between IQ and CBCL found on the Thought Problems subscale only; Klein-Tasman et al. [26] As such, the relationship between cognitive functioning and Internalising and Externalising Problems may change over time and present differently between males and females. However, given the inconsistencies in the literature, more research is required to investigate the influence of sex, chronological age, and IQ on CBCL profiles over time, especially in young WS children.

### 1.3. Family Environment and Internalising and Externalising Symptoms

The association between family factors, such as the family environment (e.g., level of conflict and cohesion), and psychopathological symptoms has received little attention in the WS literature. In typically developing school-aged children, longitudinal and cross-sectional research investigating the association between the family environment and children’s Internalising and Externalising Problems has often identified significant relationships between the family environment (especially higher conflict, lower cohesion) and higher Internalising and Externalising Problems [4,5,6]. Further, this association appears to be moderated by the child’s chronological age, suggesting that younger children are particularly vulnerable to the effects of the family environment on psychopathology outcomes; however, the directionality between the quality of the family environment and children’s Internalising and Externalising Problems requires further consideration [5]. Brawn and Porter [28] investigated the family environment in older children and adults with WS (aged 6 to 39 years) using the Family Environment Scale (FES; [29]) and identified that the family environment was characterised by high family cohesion and support and low conflict. Further, of the ten family environment subscales on the FES, only low independence (i.e., decreased tendency to make one’s own decision in the family) was significantly associated with maladaptive externalising behaviours in WS using the Vineland Adaptive Behaviour Scales, Third Edition (Vineland-III; [28]). The association between family environment and CBCL profiles has not been investigated in the WS population; however, the relationship between CBCL profiles and family stress has been considered. Research investigating stress in WS families has found that WS parents report significantly higher levels of stress than parents of preschool- and school-aged children with other genetic and neurodevelopmental conditions (e.g., Down Syndrome) and typically developing controls [2,20]. Family stress is, in turn, significantly associated with increased Externalising Problems (particularly with WS children of a younger age) and may be inversely correlated with Internalising Problems (i.e., higher Internalising Problems are associated with lower family stress); however, this finding is inconsistent between studies [3,20]. To guide support services and inform intervention, future research is needed to explore the relationship between the family environment and psychopathological symptomology in WS children.

### 1.4. The Current Study

In light of the above, the present study had two primary aims. The first primary aim was to investigate the longitudinal trajectory of CBCL profiles of young WS children and typically developing (TD) children aged 2–7 years. It was hypothesised that elevated Internalising and Externalising Problems would be present in a large proportion of young WS children and would remain chronic over a 2.5-year period, consistent with Einfeld et al. [15,16] and Woodruff-Borden et al. [14]. A sub-aim was to compare the CBCL profiles in young WS children with TD controls and investigate the associations between CBCL subscales, demographic variables (sex, chronological age), and cognitive ability (IQ/developmental quotient). It was hypothesised that WS children would present with significantly elevated Total, Internalising, and Externalising Problems compared to TD controls at both time points, consistent with the existing literature [20,22]. In line with the literature, it was further anticipated that CBCL scores in WS children would not be significantly associated with sex [22,23] or cognitive ability [19,21] but would be significantly associated with chronological age [20,22,23].

The second primary aim of the present study was to investigate the cross-sectional and longitudinal associations between the family environment at Time 1 and CBCL outcomes at both Time 1 and Time 2. In line with cross-sectional research using typically developing samples [4,5,6], it was hypothesised that CBCL outcomes would be significantly associated with family cohesion and conflict and that this association would be observed across both WS and TD controls. A sub-aim was to compare family environment profiles in WS and TD controls. Based on Brawn and Porter [28], we anticipated that the family environment would be largely comparable between groups, with the exception of lower independence for young WS families compared to the TD control group.

## 2. Method

### 2.1. Participants

The study sample consisted of 63 children and their parents/guardians (who provided consent and completed questionnaire-based measures), including young children with WS (*n* = 16; 8 males, 8 females) and a community comparison group of typically developing (TD) controls (*n* = 46; 17 males, 29 females). The WS group was recruited through Williams Syndrome Australia Limited and Williams Syndrome New Zealand. The WS diagnosis was confirmed via microarray analysis which detected the 7q11.23 microdeletion [30,31]. WS children were screened for a history of psychological, neurodevelopmental, neurological, and major sensory impairments that were unrelated to their WS, and no child was excluded from the study based on these criteria. The TD group were recruited through Macquarie University’s Neuronauts Science Club and also screened for a history of psychological, developmental, and neurological conditions, as well as major sensory impairments. One TD child was excluded from the study due to scores within the mild intellectual disability range. Data were collected between 2014 to 2019, and, as such, were not impacted by the COVID-19 pandemic.

Descriptive and demographic data of both groups at Time 1 and Time 2 are shown in Table 1. The average time between Time 1 and Time 2 was 2 years and 6 months (SD = 0.53, range 1.7–3.9). Depending on chronological age at Time 1, cognitive ability for WS and TD controls was evaluated using either the Mullen Scales of Early Learning (MSEL; [32]) or the Differential Ability Scales, Second Edition (DAS-II; [33]). The overall developmental quotients (DQs) of WS children ranged from 29.36 (severely impaired) to 69.13 (mildly impaired) (*M* = 54.47, *SD* = 10.89), which, consistent with the literature [1], suggested a mild-to-moderate range of disability. The DQs of TD controls ranged from 86.14 (low average) to 135.01 (very superior) (*M* = 107.21, *SD* = 11.72), which was in keeping with a neurotypical sample. Socioeconomic status (SES) was assessed using the Australian Bureau of Statistics (ABS) Index of Relative Socio-economic Advantage and Disadvantage (ABS; Australian Bureau of Statistics, 2016) and families were assigned a score from “1” (most socioeconomic disadvantage) to “5” (least socioeconomic disadvantage). The WS group showed significantly lower SES scores (*M* = 3.42, *SD* = 1.50) compared to the TD group (*M* = 4.65, *SD* = 0.74) (*t*(60) = −4.24, *p* < 0.001). Years of education for mothers and fathers and the average parent education were also calculated. Groups were matched on chronological age at Time 1 (*t*(60) = −1.12, *p* = 0.27) and Time 2 (*t*(60) = −1.61, *p* = 0.11), as well as sex distribution (Fisher’s exact test, *p* = 0.39), mother education (*t*(59) = −1.32, *p* = 0.19), father education (*t*(59) = −1.59, *p* = 0.11), and average parent education (*t*(59) = −1.84, *p* = 0.07).

SES, mothers’ total years of education, fathers’ total years of education, and average parent education were not significantly associated with any CBCL subscales at Time 1 for both the WS and TD groups. For SES at Time 2, mothers’ total years of education, fathers’ total years of education, and average parent education were not significantly associated with any CBCL subscales at Time 2 in either group, with the exception of a significant positive association between SES and aggressive behaviour in the TD group. SES, mothers’ total years of education, fathers’ total years of education, and average parent education were not significantly associated with FES subscales in the WS group. In the TD group, in the FES subscales, significant positive associations were found between the following: (1) mothers’ total years of education, independence, and Intellectual Cultural Orientation; (2) fathers’ total years of education and Moral Religious Emphasis; and (3) average parent education, Intellectual Cultural Orientation, and Moral Religious Emphasis. See Appendix A for tabulated correlational data.

### 2.2. Measures

#### 2.2.1. Child Behaviour Checklist for Children Ages 1.5–5 (CBCL/1.5–5) and Child Behaviour Checklist for Children Ages 6–18 (CBCL/6–18) [24]

The CBCL/1.5–5 and CBCL/6–18 are amongst the most commonly used assessment tools to measure psychopathology and behaviour impairments in children. Both CBCL versions consist of 100 items that map onto empirically based/syndrome scales and DSM-oriented scales. The empirically based/syndrome scales are summed to calculate overall summary scales, including Total Problems, Internalising Problems and Externalising Problems. Parents/guardians rate the child’s behaviour and emotional functioning at the time of testing (or within the last two-month period) on a three-point Likert scale (0 = Not True, 1 = Somewhat or Sometimes True, and 2 = Very True or Often True). Raw scores are converted to *T* scores (*M* = 50, *SD* = 10). Scores are transformed to standard scores separately for each age group and gender. Classification of *T* scores for the summary scales are as follows: <59 = normal range, 60–63 = borderline range, and >64 = clinical range. Classification of *T* scores for the syndrome and DSM-orientated scales are as follows: <64 = normal range, 65–69 = borderline range, and >70 = clinical range. The CBCL/1.5–5 and CBCL/6–18 have excellent test–retest reliability (ranging from 0.93 to 0.89) and internal consistency for subscales and composite scales (ranging from 0.62 to 0.92), as well as supported construct and criterion-related validity [24]. Both versions of the CBCL have been reliably used in WS samples with the preschool and school age range [19,21,27].

#### 2.2.2. Combining the CBCL/1.5–5 and CBCL/6–18

The CBCL/1.5–5 and CBCL/6–18 were administered across Time 1 (*n* = 49; *n* = 13, respectively) and Time 2 (*n* = 18; *n* = 44, respectively), depending on the child’s chronological age. The same primary caregiver (parent/guardian) completed the CBCL at both time points. The subscales of the CBCL/1.5–5 and CBCL/6–18 were designed to be directly comparable in order to facilitate continuity in clinical practice and for the purpose of longitudinal research [24] As such, the CBCL/1.5–5 and CBCL/6–18 were merged for the statistical analysis and will be referred to jointly as the CBCL from this point on. This process is in line with other published research assessing CBCL profiles in young WS children (e.g., [19]).

#### 2.2.3. The Family Environment Scale (FES; [29])

The FES is a measure of the social environment in families which has been used to assess the role of family coping at times of crisis and life transitions. It consists of 90 true–false items which map onto 10 subscales and three broad dimensions, including the following: (1) Family Relationship (Cohesion, Expressiveness, Conflict), (2) Personal Growth (Independence, Achievement Orientation, Intellectual Cultural Orientation, Active-Recreational Orientation, Moral Religious Emphasis), and (3) System Maintenance and Change (Organisation, Control). Raw scores are converted to *T* scores (*M* = 50, *SD* = 10), and a *T* score ≥ 60 is considered elevated. The FES has sound reliability, with test–retest reliability ranging from 0.68 to 86 and internal consistency reliabilities ranging from 0.61 to 0.78 [29].

#### 2.2.4. The Mullen Scales of Early Learning (MSEL; [32])

The MSEL [32] is a standardised assessment of early cognitive functioning in infants and preschool-aged children, ranging from birth to 68 months. It consists of four subtests, including the following: Visual Reception, Fine Motor, Receptive Language, and Expressive Language. Raw scores for each subtest are converted to *T* scores (*M* = 50, *SD* = 10), with a lower scoring indicating greater cognitive difficulties. An overall measure of cognition, the Early Learning Composite, is also calculated and reported as a standard score (*M* = 100, *SD* = 15). Internal consistency has been established for the Early Learning Composite (ranging from 0.83 to 0.95) and subtest (ranging from 0.75 to 0.83) (Mullen, 1995). Interscorer reliability (ranging from 0.91 to 0.99) and test–retest reliability (ranging from 0.71 to 0.96) have also been reported [32]). Reliability and validity within the WS population have been established in previous studies (e.g., [35]).

#### 2.2.5. Differential Ability Scales, Second Edition (DAS-II; [33])

The DAS-II [33] is a standardised assessment of intellectual functioning in children aged from 2 years, 6 months to 17 years, 11 months. It consists of the Early Years battery (for children aged 2 years, 6 months to 6 years, 11 months) and School-Aged battery (for children aged 7 to 17 years, 11 months). The low level of the Early Years battery consists of four core subtests which map onto an overall General Conceptual Ability (GCA) score, as well as two cluster scores (Verbal Ability and Nonverbal Ability). The upper-level Early Years and School-Aged batteries consist of six core subtests which map onto the GCA, as well as three cluster scores (Verbal Ability, Nonverbal Reasoning Ability, and Spatial Ability). Raw scores for each subtest are converted to a *T* score (*M* = 50, *SD* = 10), and a standard score for the GCA and each cluster is also calculated (*M* = 100, *SD* = 15). Internal reliability has been established for each subtest, cluster, and GCA for both the Early Years battery (ranging from 0.82 to 0.94) and School-Aged battery (ranging from 0.68 to 0.97) [33]. Construct and concurrent validity are also supported [33]. Reliability and validity of the DAS-II within the WS population have been established in previous studies (e.g., [36]).

#### 2.2.6. Combining MSEL and DAS-II Scores

Each child’s intellectual functioning was assessed at Time 1 to yield three overall scores: Global (i.e., overall DQ), Verbal, and Nonverbal. These scores were calculated using with the MSEL or DAS-II depending on the child’s age at the time of testing. DQ scores were manually calculated using the MSEL four core subtests to maintain consistency with the DAS-II GAC, Verbal, and Nonverbal scores. The calculated MSEL DQ scores (all children under 68 months of age at Time 1; WS: *n* = 13; TD: *n* = 32) and the DAS-II verbal cluster, nonverbal cluster, and GAC scores (all children over 68 months of age at Time 1; WS: *n* = 3; TD: *n* = 14) were combined to create single measures of Global, Verbal, and Nonverbal ability for each WS and TD child, in order to reduce the probability of making a Type-II error. This process is aligned with other published research assessing young children with neurodevelopmental disorders, including WS populations (e.g., [37]).

### 2.3. Procedure

Ethics approval for this study was gained from the Macquarie University Human Research Ethics Committee (reference numbers: 5200900071 and 52021913524613). The MSEL and DAS-II were administered to the children in accordance with standardised instructions (Mullen, 1995; Elliot, 2007 [32,33]). The preschool- and school-aged CBCL measures and FES were administered to the parents/caregivers on the same day as the cognitive testing. The CBCL, FES, MSEL, and DAS-II were scored by hand according to each of the examiner’s manuals and then checked with their respective computer scoring programs, with the exception of FES which was double-scored by hand [24,29,32,33].

### 2.4. Analytic Approach

The statistical analysis was conducted using the IBM SPSS Statistics version 25. A *p* value of 0.01 was used to minimise the possibility of making both Type-I and Type-II errors during multiple comparisons and to maintain consistency with published studies on WS (e.g., [21,22]). G*Power software (https://www.psychologie.hhu.de/arbeitsgruppen/allgemeine-psychologie-und-arbeitspsychologie/gpower) was used to conduct a post hoc power analysis [38] to test the difference between two independent group means and paired group means using two-tailed tests, a large effect size (*d* = 0.9), and an alpha of 0.01. For the independent sample *t*-tests, results showed that an unequal sample size between groups of *n* = 16 and *n* = 46 would achieve an adequate power of 0.70. For the paired sample *t*-tests, results showed that a total sample size of *n* = 62 would achieve an adequate power of 0.99. A post hoc correlation power analysis was also conducted using a correlation of 0.39 [4,7], an alpha of 0.01, and a sample size of *n* = 62, and the results indicated an achieved power of 0.73. As such, the sample obtained provided adequate statistical power for the paired sample *t*-tests; however, the power was low for independent sample *t*-tests and correlations. Given the small sample size and subsequent low power, linear regression analyses were not used. Effect sizes for *r* were classified as the following: ≤0.1 = small; 0.3 = medium; and ≥0.0.5 = large. Effect sizes for *d* were classified as the following: ≤0.2 = small; 0.5 = medium; and ≥0.0.8 = large [39]. Nonparametric tests were utilised when analysing data that violated assumptions, which included the following CBCL subscales: Anxious/Depressed (Time 1), Withdrawn/Depressed (Time 1, Time 2), Somatic Complaints (Time 1, Time 2), Attention Problems (Time 1, Time 2), Aggressive Behaviours (Time 1, Time 2), Affective Problems (Time 1, Time 2), Attention-Deficit Hyperactivity Problems (Time 1, Time 2), and Oppositional Defiant Problems (Time 1, Time 2).

In the longitudinal analyses, paired sample *t*-tests were used to compare all CBCL mean *T* scores at Time 1 and Time 2, for both WS and TD controls. To investigate reliable change, the difference between CBCL *T* scores for each child at Time 1 and Time 2 was calculated and reliable change was determined when the difference exceeded one standard deviation of the normative sample [24].

In the profile analyses, the descriptive statistics of CBCL profiles were analysed at the group and individual level. Independent sample *t*-tests were employed to compare the mean *T* scores of WS to TD controls across all CBCL subscales and at both time points. Stacked bar graphs and box plots were used to illustrate the distribution of the range of CBCL scores for WS and TD children at both time points. A series of correlations were then used to investigate whether CBCL scores varied according to sex, chronological age, and cognitive ability (Global DQ, Verbal DQ, Nonverbal DQ).

In the FES profile analyses, a series of correlations were used to investigate whether CBCL scores at Time 1 and 2 varied according to FES scores at Time 1. The descriptive statistics of FES profiles were analysed at the group level and independent sample *t*-tests were employed to compare the mean *T* scores of WS to TD controls across all FES subscales.

## 3. Results

Table 2 shows the mean, standard deviation, and range of *T* scores across the CBCL subscale scores for both the WS and TD groups. Paired sample *t*-tests investigating the difference between CBCL subscale scores from Time 1 to Time 2 for WS and TD children, on average, and independent sample *t*-tests investigating the difference between groups at each time point are also reported in Table 2.

### 3.1. Longitudinal Trajectory of CBCL Profiles in WS and TD Controls

In the longitudinal analysis, paired sample *t*-tests revealed no significant change in CBCL scores between Time 1 and Time 2 in WS or TD children, on average. Reliable change scores were analysed to investigate change in CBCL scores over time at an individual rather than a group level. Table 3 illustrates reliable change scores for both WS and TD controls. Among the WS group, there were reliable changes for a large number of WS children. The Attention Problems subscale showed the highest percentage of children with a reliable increase in their scores over time (50%), followed by the Total Problems summary subscale (43.5%) and Aggressive Behaviour subscale (37.5%). A significant decrease in scores was most commonly seen for Externalising Problems (75%), followed by Withdrawn/Depressed (31.25%), Attention-Deficit Hyperactivity Problems (25%), and Oppositional Defiant Problems (25%). The majority of WS children did not show reliable change over time in the following subscales: Internalising Problems (68.75%), Anxious/Depressed (56.25%), Somatic Complaints (62.5%), Affective Problems (62.5%), and Oppositional Defiant Problems (68.75%). In contrast to WS, as can be seen in Table 3, the majority of TD children did not show reliable change over time across all CBCL subscales. TD controls showed the highest increase in scores over time on Externalising Problems (32.6%) and Internalising Problems (30.4%). The most commonly reported decreases in change over time were also reported for these summary subscales at 17.4%.

### 3.2. CBCL Profiles in WS and TD Children at Time 1 and Time 2

**Figure 1:**
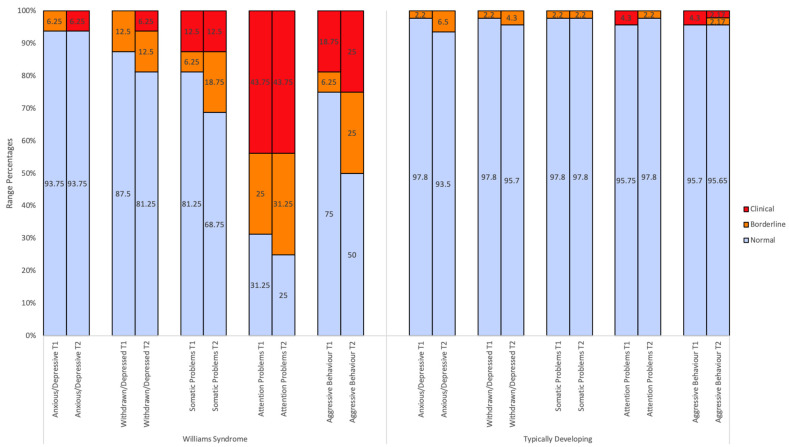
CBCL syndrome scales’ range percentages at Time 1 and Time 2. Note. T1 = Time 1. T2 = Time 2.

In the cross-sectional analysis of CBCL profiles, at the group level, Attention Problems emerged as the syndrome subscale with the highest mean *T* scores and highest percentage of children in the clinical range (43.75%; see Figure 1) at both time points for WS children. Summary scales were amongst the highest average scores reported at Time 2 in the WS group, with Externalising Problems identified with a mean *T* score of 63.44 (43.75% in the clinical range) and Total Problems identified with a mean *T* score of 62.43 (56.25% in the clinical range). This was in contrast to the CBCL profiles of TD controls, with summary scales *T* scores reported between 43 to 47 and 2.2 to 6.5% of TD children falling in the clinical range across time points.

**Figure 2:**
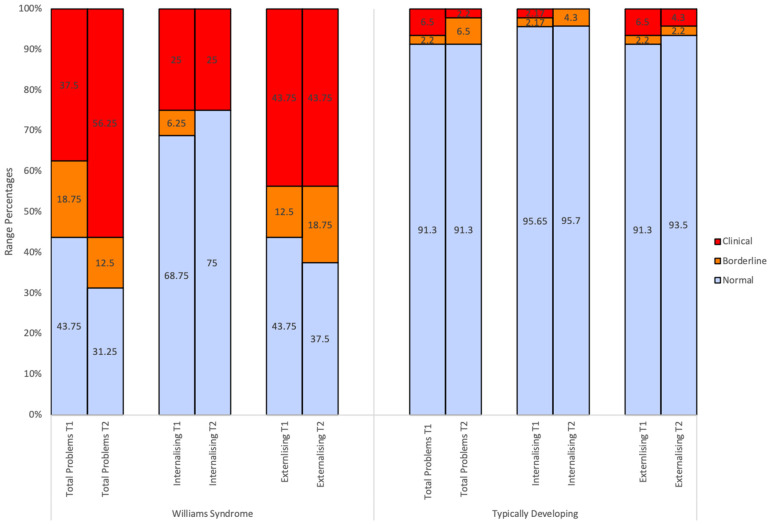
CBCL summary scales’ range percentages at Time 1 and Time 2. Note. T1 = Time 1. T2 = Time 2.

**Figure 3:**
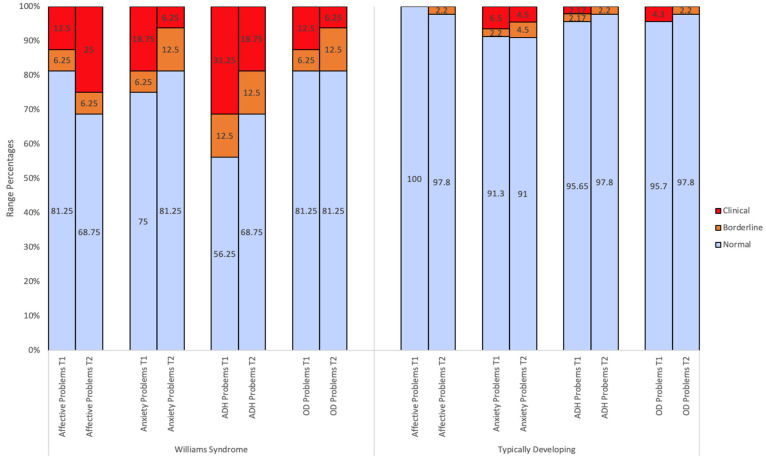
CBCL DSM-oriented scales’ range percentages at Time 1 and Time 2. Note. T1 = Time 1. T2 = Time 2. ADH = Attention-Deficit Hyperactivity. OD = Oppositional Defiant.

CBCL scores for WS and TD children have been displayed graphically to illustrate the percentage of individuals falling in each descriptive range (normal, borderline, clinical) and the distribution of scores for CBCL summary scales. Figure 1, Figure 2 and Figure 3 show the percentage of *T* scores which fell in the normal, borderline, and clinical range for WS and TD controls for each CBCL subscale. At an individual level, Table 2 and Figure 1, Figure 2 and Figure 3 illustrate that WS children display scores in the clinically significant range on all CBCL subscales at both Time 1 (with the exception of Anxious/Depressed and Withdrawn/Depressed) and Time 2.

### 3.3. Comparison of WS to TD at Time 1 and Time 2

Independent sample *t*-tests revealed that the WS group had higher mean *T* scores relative to TD controls across most CBCL subscales across both time points. At Time 1 and Time 2, the WS group displayed significantly higher mean *T* scores on the following scales: Total Problems (*t*(60) = 5.36, *p* < 0.01; *t*(60) = 6.89, *p* < 0.01), Internalising Problems (*t*(60) = 4.32, *p* < 0.01; *t*(60) = 3.61, *p* < 0.01), Externalising Problems (*t*(60) = 5.02, *p* < 0.01; *t*(60) = 6.07, *p* < 0.01), Withdrawn/Depressed (*t*(18.12) = 4.32, *p* < 0.01; *t*(18.58) = 2.92, *p* < 0.01), Attention Problems (*t*(19.18) = 5.96, *p* < 0.01; *t*(16.33) = 6.93, *p* < 0.01), Aggressive Behaviour (*t*(18.40) = 3.135, *p* = 0.01; *t*(16.70) = 3.75, *p* < 0.01), Affective Problems (*t*(17.25) = 4.64, *p* < 0.01; *t*(16.20) = 3.82, *p* < 0.01), Anxiety Problems at Time 2 (*t*(59) = 2.65, *p* = 0.01), Attention-Deficit Hyperactivity Problems (*t*(18.01) = 4.62, *p* < 0.01; *t*(17.07) = 4.31, *p* < 0.01), and Oppositional Defiant Problems at Time 2 (*t*(18.70) = 2.92, *p* < 0.01). See Table 2 for all independent sample *t*-test results. Figure 1, Figure 2 and Figure 3 further illustrate that in the WS group, larger proportions of children fell in the borderline-to-clinical ranges and the distribution of scores was more elevated compared to TD controls.

### 3.4. Correlations between Sex, Chronological Age, Cognitive Ability, and CBCL Profile

A series of Pearson and Spearman’s Rho correlation coefficients were conducted to assess the relationship between sex, chronological age, cognitive ability, and CBCL scores in WS and TD controls at both Time 1 and Time 2 (see Appendix B for tabulated correlational data). In the WS group, there was no significant relationship between sex, chronological age, Global DQ, Verbal DQ, and Nonverbal DQ on any of the CBCL scales at either time point.

In the TD control group, a medium, significant positive effect size was found between sex and Anxiety Problems at Time 2 (*r_s_*(44) = 0.39, *p* < 0.01), suggesting that female TD children displayed higher Anxiety Problems at Time 2 compared to males. A medium, significant positive effect size was found between chronological age and Anxious/Depressed at Time 1 (*r*(44) = 0.40, *p* < 0.01), indicating that Anxious/Depressed scores were higher in older children. No other CBCL scales were significantly correlated with sex, chronological age, or cognitive ability (Global DQ, Verbal DQ, Nonverbal DQ).

### 3.5. Relationship between FES and CBCL in WS and TD Controls

A series of Pearson and Spearman’s Rho correlation coefficients were conducted to assess the relationship between FES scores at Time 1 and CBCL scores at Time 1 and Time 2. See Table 4 for tabulated correlational data. In the WS group cross-sectionally at Time 1, significant large and positive effect sizes were found between FES Conflict and Attention Problems (*r_s_*(14) = 0.66, *p* < 0.01). No other significant relationships were identified in the WS group at either time point.

In the TD group cross-sectionally at Time 1, significant medium and positive effect sizes were found between FES Conflict and CBCL Total Problems (*r*(43) = 0.38, *p* = 0.01). There were significant medium and negative effect sizes between FES Active Recreational Orientation and CBCL Total Problems (*r*(43) = −0.30, *p* < 0.01) and Affective Problems (*r_s_*(42) = −0.45, *p* < 0.01). In the TD group utilising FES ratings at Time 1 and the CBCL ratings at Time 2 to investigate longitudinal associations, there were significant medium and negative effect sizes between FES Cohesion and CBCL Total Problems (*r*(43) = −0.40, *p* < 0.01) and Affective Problems (*r_s_*(42) = −0.40, *p* < 0.01). A significant large and positive effect size was identified between FES Conflict and CBCL Total Problems (*r*(43) = 0.55, *p* < 0.01) and Externalising Problems (*r*(43) = 0.53, *p* < 0.01), and significant medium, positive effect sizes were identified between FES Conflict and Affective Problems (*r_s_*(42) = 0.40, *p* < 0.01) and Oppositional Defiant Problems (*r_s_*(42) = 0.39, *p* < 0.01). Significant medium and negative effect sizes were found between FES Achievement Orientation and CBCL Externalising Problems (*r*(43) = −0.41, *p* < 0.01) and Oppositional Defiant Problems (*r_s_*(42) = −0.41, *p* < 0.01). Significant medium, negative effect sizes were also found between FES Active Recreational Orientation and CBCL Total Problems (*r*(43) = −0.41, *p* < 0.01), Internalising Problems (*r*(43) = −0.43, *p* < 0.01), and Affective Problems (*r_s_*(42) = −0.40, *p* < 0.01), as well as between FES Moral Religious Emphasis and CBCL Internalising Problems (*r*(43) = −0.41, *p* < 0.01). Lastly, significant medium, negative effect sizes were found between FES Organisation and CBCL Externalising Problems (*r*(43) = −0.38, *p* = 0.01) and Oppositional Defiant Problems (*r_s_*(42) = −0.46, *p* < 0.01). No other significant relationships were identified.

### 3.6. FES Profile in WS and TD Children at Time 1

Table 5 shows the mean, standard deviation, and range of *T* scores across the FES subscales for each group, as well as results of independent sample *t*-tests. Independent sample *t*-tests revealed that the WS group had significantly lower average Intellectual Cultural Orientation scores compared to TD controls (*t*(59)=-3.33, *p* < 0.01). There were no significant differences between WS and TD scores on the remaining FES subscales.

## 4. Discussion

This was the first known study to explore the trajectory of internalising and externalising profiles during early development in WS children using the CBCL and to investigate the impact of the family environment on psychopathology symptomology. Whilst demographic variables have been considered in the existing literature [19,20,21,22,23], the impact of the family environment has received little research [28] and, prior to this study, none of the known literature has explored whether differences in the family environment exist between WS families and families of typically developing children. As such, the present study sought to achieve two primary aims. The first aim was to investigate the longitudinal trajectory of internalising and externalising profiles in young WS children, with the sub-aims of cross-sectionally exploring the differences between WS children and TD controls on the CBCL subscales and investigating the association between CBCL subscale scores, demographic variables, and cognitive ability. The second aim was to investigate longitudinal and cross-sectional associations between the family environment and internalising and externalising outcomes in young WS children compared to TD controls, with the sub-aim of profiling the family environment in WS compared to TD families.

### 4.1. Longitudinal Trajectory of CBCL Profiles

This was the first known study to investigate the longitudinal trajectory of CBCL profiles in young WS children and the hypothesis that CBCL scores would remain consistently elevated over time was supported (in line with [14,15,16].The longitudinal analysis revealed that there was no significant difference in CBCL profiles in WS children between time points, on average, which suggests that at the group level, internalising and externalising problems remained chronically elevated over time, in line with expectations [14,15,16,20,22,23]. However, at the individual level, WS children demonstrated considerable variability in the change in their CBCL scores over time. A considerable portion of WS children showed significant increases or decreases in CBCL scores, and the fluctuation of scores in both directions may account for the absence of significant differences in average scores on CBCL subscales over time. This was in contrast to TD controls who typically did not show a clinically meaningful change between time points at either the group or individual level. Future research is needed to investigate which factors predict both increases and decreases in internalising and externalising problems over time in young WS children. Such research would be valuable in informing the most salient factors to target in early intervention.

### 4.2. CBCL Profiles and Associations with Demographic Variables and Cognitive Ability

Consistent with the existing literature [3,20,22] and in line with expectations, WS children showed significantly elevated internalising and externalising problems when compared to TD controls. Overall, over half of the WS cohort showed elevated Total Problems and Externalising Problems on the CBCL at both time points. Externalising Problems were characterised by elevated Attention Problems (which were reported in approximately three-quarters of WS children), Aggressive Behaviour (reported in one-quarter to half of WS children), and Attention-Deficit Hyperactivity Problems (reported in one-third to half of WS children). In line with the previous literature [3,20,22], Internalising Problems in WS children were also significantly higher than TD children and were characterised by elevated Affective Problems (19–31% in the borderline-to-clinical range) and Anxiety Problems (19–25% in the borderline-to-clinical range).

In profiling the CBCL, this study also investigated the association between CBCL profiles and demographic variables. In line with expectations [19,21,22], the CBCL subscales at both time points were not significantly associated with sex or developmental/intellectual ability in WS children. Contrary to expectations [20,22,23], however, chronological age in WS children was not significantly associated with CBCL subscales at this young age.

### 4.3. Relationship between the Family Environment and Psychopathology Symptomology

The second primary aim of this study was to analyse the impact of the family environment at Time 1 on internalising and externalising outcomes at Time 1 and Time 2 in young WS children and TD controls. Overall, there was a general trend of a greater number of significant correlations between FES and CBCL subscales in the TD group compared to the WS group at both time points. Specifically, in WS children, conflict in the family environment was only significantly associated with Attention Problems at Time 1, with no other significant correlations identified at either time point. This was in contrast to TD families, in which conflict in the family environment was significantly positively associated with higher Total Problems (at both time points) and with Externalising Problems, Affective Problems, and Oppositional Defiant Problems (at Time 2), which was in line with the previous literature using TD children [4,5,6]. Contrary to expectations [4,5,6], however, family conflict was not significantly associated with overall Internalising Problems in either group. Further, the expected association between family cohesion and psychopathology symptomology (Total Problems) was only significant in TD families at Time 2 and did not reach statistical significance in WS families at either time point.

Overall, it is notable that fewer significant associations were found between the family environment and psychopathological outcomes in young WS children compared to TD controls. Whilst the directionality of these associations is unknown, it could be hypothesised that WS children may be less susceptible to the impact of the family environment on internalising and externalising outcomes than TD children. As such, it could be inferred that elevated CBCL scores in WS children are better explained by underlying mechanisms (i.e., neuroanatomical differences caused by the microdeletion that are associated with internalising and externalising problems; [40,41,42,43], as opposed to a causative impact of environmental factors.

### 4.4. Profile of the Family Environment in Families of WS and TD Children

The profile of the family environment in young WS children compared to TD controls was also explored. This study hypothesised that the family environment would be comparable between groups, with the exception of lower independence in WS families compared to TD controls [28]. This hypothesis was partially supported. WS and TD families reported similar scores on the FES and there was an absence of significantly different scores between groups on nine of ten subscales. Contrary to expectations based on previous research [28]), no statistically significant difference was found between families with young WS and TD children on the Independence subscale. These WS and TD families only differed significantly in the Intellectual Cultural Orientation subscale, which measures interest in intellectual, cultural, and political activities (e.g., attending concerts, discussing politics). It is probable that differences between studies reflect the age of the samples, with Brawn and Porter [28] using an older sample spanning from children to adults (6 to 39 years) and the present study using an early childhood sample. Previous qualitative research conducted with WS families sheds further light on these findings, with WS parents noting concerns regarding independence only as WS children transition into adulthood [44], suggesting that independence is a primary concern in older, as opposed to younger, WS age groups. Qualitative research further notes frequent experiences of restricted daily living activities in WS families [44], which may speak to the lower score on the Intellectual Cultural Orientation subscale in WS compared to TD families in the current study. Future research should explore the changing needs of WS families over the lifespan to best direct support services for the family unit.

### 4.5. Practical Implications

This study highlights that elevated internalising and externalising problems are evident in very young WS children and that these problems, on average, are chronic over time. Despite this, WS children also show more fluctuation in internalising and externalising problems at the individual level when compared to TD children. WS families should be supported to access services that would aid in early identification, monitoring, and individualised management of problematic emotional and behavioural functioning, including targeted early intervention that provides psychoeducation on the chronic course and fluctuating nature of psychopathology concerns over the lifespan of WS individuals. Intervention and management of elevated internalising and externalising problems should include parents as well as educators to address the functional impact of such difficulties and aid with academic planning (an area of concern for WS parents; [44]). Moreover, environmental and biological factors that may relate to fluctuations in psychopathological symptoms are important clinical and research considerations, and parent/guardian concerns regarding the safety of facilitating independence are also in need of both research and clinical input.

### 4.6. Limitations and Future Research

Whilst this study addressed important gaps in the current literature on WS, limitations and recommendations for future research should be considered. Firstly, the small sample size of WS children (*n* = 16), although typical of research conducted within this population, limited the applicability of statistical analyses that could inform the directionality of effects, and the low power limited sensitivity in detecting effects. It is also possible that the low power of the current study could account for the absence of significant results (e.g., between CBCL scales and chronological age). Future research utilising data across different research groups allowing for larger sample sizes would provide more robust results. Further, this study relied on parent report ratings, which increases the potential of bias. Future research could be improved by incorporating multiple informants who observe the child across environments (e.g., parents, teachers, and health care professionals). Lastly, whilst the CBCL is routinely administered in clinical practice and frequently utilised in research with the WS population, it is a screening, as opposed to diagnostic, tool. The sensitivity and specificity of this measure in detecting psychopathology outcomes may be limited in children with genetic and neurodevelopmental syndromes, particularly for DSM-oriented subscales [22,45]. Future research would benefit from incorporating a broader breadth of measures to provide a more thorough assessment of internalising and externalising problems (e.g., clinical interviews and observations). The changing needs in the family environment of WS individuals across the lifespan also warrant further investigation.

## 5. Conclusions

The current study contributes to the body of the literature conducted with WS populations by examining the early longitudinal trajectory of internalising and externalising problems during early development and the relationship with the family environment. This study highlights that elevated internalising and externalising problems are evident early on in WS individuals and remain chronically elevated, on average, over time. This study was also unique in that it explored the impact of the family environment on internalising and externalising problems in a young WS population, with results suggesting that the psychopathology outcomes of WS children are less frequently associated with the family environment than in TD controls. As such, it could be inferred that elevated psychopathology in WS may be more heavily influenced by underlying biological mechanisms (e.g., neuroanatomical alterations caused by the microdeletion). However, future research is required to further investigate this association and the relative contributions of and interactions between biological and environmental factors.

## Figures and Tables

**Table 1:** Between-group comparisons of descriptive and demographic data at Time 1 and Time 2.

Measure	WS (*n* = 16)				TD (*n* = 46)			
	Time Point							
	Time 1		Time 2		Time 1		Time 2	
	Mean (*SD*)	Range	Mean (*SD*)	Range	Mean (*SD*)	Range	Mean (*SD*)	Range
CA (years) ^a^	4.28 (1.58)	2.17–6.92	6.62 (1.83)	3.98–9.48	4.82 (1.67)	2.23–7.86	7.45 (1.75)	4.87–11.12
Overall DQ ^b^/IQ ^c^	54.47 (19.89)	29.36–69.13	-	-	107.21 (11.72)	86.14–135.01	-	-
Verbal DQ ^b^/IQ ^c^	57.12 (15.95)	23.71–93	-	-	107.48 (11.51)	85–142	-	-
Nonverbal DQ ^b^/IQ ^c^	58.96 (11.83)	35–75	-	-	105.69 (13.88)	84.49–148	-	-
Family SES ^d^	3.43 (1.50)	1–5	3.43 (1.50)	1–5	4.65 (0.74)	2–5	4.52 (0.91)	2–5
Years of Education								
Mother	14.13 (2.10)	10–17			14.91 (1.95)	12–18		
Father	13.00 (2.33)	9–17			14.07 (2.23)	10–18		
Average Parent Years of Education	13.57 (1.88)	9.50–16			14.49 (1.62)	11–18		
	*n*	%			*n*	%		
Gender distribution	8 M 8 F	50 50			17 M 29 F	37 63		
Family Ethnicity								
% Oceanian	42				44			
% European	17				20			
% Asian	0				5			
% Middle Eastern	8				0			
% Other/Mix ^e^	33				31			

Note. ^a^ chronological age. ^b^ DQ = developmental quotient as measured by MSEL or DAS-II at Time 1 (Standard Scores; *M* = 100, *SD* = 15). ^c^ IQ = intelligence quotient as measured by DAS-II at Time 2 (Standard Scores; *M* = 100, *SD* = 15). ^d^ SES = socioeconomic status as measured by the Australian Bureau of Statistics Index of Relative Socio-economic Advantage and Disadvantage (ABS; [34] based on geographic residence. Families were provided with a score from “1” to “5”, with a lower score denoting a greater socioeconomic disadvantage. ^e^ Other/Mix = multiple ethnicities identified, including Oceanian, European, North African, Middle Eastern, Asian, and Australian.

**Table 2:** CBCL data, paired sample *t*-tests, and independent sample *t*-tests for WS and TD at Time 1 and Time 2.

CBCL Subscales	WS							TD							Between-Group Comparisons (WS vs. TD) ^b^					
	Time 1		Time 2		Change over Time (WS T1 vs. T2) ^a^			Time 1		Time 2		Change over Time (TD T1 vs. T2) ^a^			Time 1			Time 2		
	Mean (*SD*)	Range	Mean (*SD*)	Range	*T* (*15*)	*p*	*d*	Mean (*SD*)	Range	Mean (*SD*)	Range	*T* (*45*)	*p*	*d*	*T* (*df*)	*p*	*d*	*T* (*df*)	*p*	*d*
Summary Scales																				
Total Problems	59 (11.69)	37–**77**	62.43 (10.97)	37–**77**	−1.12	0.28	−0.28	44.67 (8.07)	28–**64**	43 (9.78)	28–**68**	−1.22	0.23	−0.18	5.36 (60)	<0.01	1.56	6.89 (60)	<0.01	2.00
Internalising Problems	56.06 (9.48)	33–**71**	56.43 (10.06)	33–**72**	−1.15	0.89	−0.04	47.07 (8.56)	29–61	43.65 (10.03)	29–**65**	−2.10	0.04	−0.31	4.31 (60)	<0.01	1.25	3.61 (60)	<0.01	1.05
Externalising Problems	59.06 (12.02)	40–**80**	**63.44** (9.24)	47–**76**	−1.51	0.15	−0.38	45.57 (9.68)	28–**67**	43.30 (10.39)	28–**76**	−1.32	0.19	−0.20	5.02 (60)	<0.01	1.46	6.07 (60)	<0.01	1.76
Syndrome Scales																				
Anxious/Depressed	54.19 (5.04)	50–66	56.44 (7.14)	50–**78**	−1.12	0.28	−0.28	52.41 (4.14)	50–66	51.93 (3.81)	50–66	−0.75	0.46	−0.11	1.87 (60)	0.07	0.54	2.13 (18.63)	0.05	0.80
Withdrawn/Depressed ^c^	57.75 (5.97)	50–67	57.63 (7.09)	50–**73**	0.07	0.94	0.02	52.15 (4.08)	50–68	51.87 (3.22)	50–66	−0.47	0.64	−0.07	3.75 (18.12)	<0.01	1.44	2.92 (18.58)	<0.01	1.09
Somatic Complaints ^c^	57.56 (7.22)	50–**72**	57.81 (7.54)	50–**72**	−0.12	0.91	−0.03	53.72 (4.44)	50–68	52.46 (4.72)	50–65	−1.40	0.17	−0.21	2.64 (19.64)	0.02	0.94	2.05 (18.74)	0.05	0.76
Attention Problems ^c^	65.44 (8.52)	51–**77**	**69.13** (9.72)	52–**90**	−1.36	0.20	−0.34	51.93 (3.45)	50–68	51.91 (5.29)	50–**77**	−0.03	0.97	−0.01	5.96 (19.18)	<0.01	2.16	6.93 (16.34)	<0.01	3.01
Aggressive Behaviour ^c^	59.75 (9.10)	50–**79**	62.31 (10.48)	50–**84**	−0.189	0.25	−0.30	52.22 (4.19)	50–**69**	52.24 (5.11)	50–**72**	0.03	0.97	0.01	3.14 (18.40)	<0.01	1.18	3.75 (16.70)	<0.01	1.58
DSM-Orientated Scales																				
Affective Problems ^c^	60.19 (6.68)	50–**75**	61.81 (10.26)	50–**82**	−0.76	0.46	−0.29	51.82 (3.42)	50–66	52.17 (3.06)	50–63	0.67	0.51	0.10	4.64 (17.25)	<0.01	1.88	3.82 (16.20)	<0.01	1.68
Anxiety Problems	57.75 (8.21)	50–**73**	58.31 (8.28)	50–**80**	−0.22	0.83	−0.06	53.24 (5.87)	50–**73**	53.5 (6.25)	50–**73**	0.39	0.70	0.06	2.16 (60)	0.04	0.63	2.65 (59)	<0.01	0.77
ADH Problems ^c^	62.13 (8.90)	50–**76**	62.13 (9.14)	50–**80**	0.11	0.91	0.03	51.96 (3.99)	50–67	51.61 (4.71)	50–**76**	−0.45	0.65	−0.07	4.62 (18.01)	<0.01	1.78	4.31 (17.07)	<0.01	1.77
OD Problems ^c^	59.31 (8.87)	50–**80**	57.63 (6.54)	50–**69**	1.08	0.30	0.27	52.58 (3.79)	50–67	52.93 (5.49)	50–**73**	0.60	0.55	0.09	2.70 (19.15)	0.01	0.98	2.92 (18.70)	<0.01	1.09

Note. Bold font indicates clinically significant group *T* scores (≥63 for summary scales; ≥69 for syndrome and DSM-orientated scales). ^a^ Paired *t*-test results. ^b^ Independent sample *t*-tests. ^c^ Scores represent Spearman’s Rho correlation coefficient. AD Problems = Attention-Deficit Hyperactivity Problems. OD Problems = Oppositional Defiant Problems.

**Table 3:** CBCL reliable change data for WS and TD.

Scales	WS (*n* = 16)			TD (*n* = 46)		
	% Increase	% Decrease	% No Change	% Increase	% Decrease	% No Change
Summary						
Total Problems	43.50	12.50	43.50	19.60	15.20	65.20
Internalising Problems	12.50	18.75	68.75	30.40	17.40	52.20
Externalising Problems	25.00	75.00	0.00	32.60	17.40	50.00
Syndrome						
Anxious/Depressed	31.25	12.50	56.25	13.00	8.70	78.30
Withdrawn/Depressed	18.75	31.25	50.00	10.90	8.70	80.40
Somatic Complaints	18.75	18.75	62.50	19.60	10.90	69.60
Attention Problems	50.00	18.75	31.25	2.20	4.30	93.50
Aggressive Behaviour	37.5.0	18.75	43.75	8.70	4.30	87.00
DSM-Orientated						
Affective Problems	18.75	18.75	62.5	6.70	6.70	86.70
Anxiety Problems	31.25	18.75	50.00	13.30	11.10	75.60
Attention-Deficit Hyperactivity Problems	31.25	25.00	43.75	13.30	6.70	80.00
Oppositional Defiant Problems	6.25	25.00	68.75	4.40	8.90	86.70

**Table 4:** FES Time 1 and CBCL Time 1 and Time 2 correlations for WS and TD sample.

CBCL Subscales	WS										TD									
	FES Time 1										FES Time 1									
	Coh	Exp	Conf	Ind	AO	ICO	ARO	MRE	Org	Cont	Coh	Exp	Conf	Ind	AO	ICO	ARO	MRE	Org	Cont
**Time 1**																				
Summary Scales																				
Total Problems	−0.03	−0.23	0.51	−0.36	0.00	−0.09	−0.21	0.45	−0.05	0.32	−0.09	0.11	0.38 **	−0.04	−0.01	−0.22	−0.39 **	0.16	−0.08	0.18
Internalising Problems	0.31	0.24	0.29	−0.17	−0.04	0.06	0.16	0.26	0.06	0.24	−0.04	0.09	0.23	0.00	−0.01	−0.11	−0.31	0.11	0.15	0.06
Externalising Problems	−0.10	−0.30	0.56	−0.22	0.01	−0.15	−0.16	0.36	0.02	0.38	0.00	0.20	0.38	−0.03	0.01	−0.20	−0.31	0.14	−0.07	0.23
Syndrome Scales																				
Anxious/Depressed	0.32	0.09	0.31	−0.22	0.04	−0.02	0.08	0.25	0.13	0.18	−0.17	−0.02	0.30	−0.09	0.08	−0.21	−0.15	−0.04	0.04	0.15
Withdrawn/Depressed ^a^	0.14	0.13	−0.06	0.14	−0.20	−0.12	0.14	−0.26	−0.04	−0.12	0.00	0.18	0.16	0.03	−0.28	−0.02	−0.14	0.08	0.04	0.01
Somatic Complaints ^a^	0.16	0.61	0.20	−0.08	0.08	0.02	0.33	−0.17	−0.21	−0.12	−0.11	−0.01	0.25	−0.08	0.03	−0.09	−0.25	0.15	0.13	−0.00
Attention Problems ^a^	−0.11	−0.50	0.66 **	−0.15	0.10	−0.40	−0.40	0.30	0.19	0.33	−0.15	−0.06	0.29	−0.15	−0.11	−0.17	−0.20	−0.00	−0.06	0.19
Aggressive Behaviour ^a^	0.02	−0.33	0.49	−0.26	0.04	−0.15	−0.17	0.41	0.01	0.27	−0.13	0.18	0.30	−0.11	−0.06	−0.23	−0.24	0.06	−0.05	0.31
DSM-Orientated Scales																				
Affective Problems ^a^	0.18	0.04	0.55	−0.44	0.01	0.01	−0.17	0.21	−0.07	0.18	−0.23	−0.06	0.27	−0.00	0.06	−0.23	−0.45 **	0.05	−0.15	0.35
Anxiety Problems	0.42	0.27	0.35	−0.30	−0.10	−0.12	0.12	0.01	0.02	−0.03	−0.18	−0.01	0.24	−0.01	0.16	−0.16	−0.21	−0.09	0.09	0.16
ADH Problems ^a^	−0.12	−0.38	0.39	−0.20	−0.02	−0.33	−0.22	0.56	0.13	0.41	−0.18	0.10	0.27	−0.20	−0.14	−0.19	−0.269	.12	−0.19	0.04
OD Problems ^a^	0.08	−0.21	0.54	−0.24	0.05	−0.20	−0.09	0.33	−0.08	0.29	−0.00	0.29	0.18	−0.03	−0.04	−0.04	−0.11	.14	−0.04	0.17
**Time 2**																				
Summary Scales																				
Total Problems	−0.29	0.07	0.49	−0.04	0.26	−0.21	−0.12	−0.08	−0.48	−0.27	−0.40 **	−0.26	0.57 **	−0.35	−0.36	−0.16	−0.41 **	−2.8	−0.33	−0.00
Internalising Problems	−0.05	0.07	0.12	−0.32	0.12	−0.21	−0.30	0.40	−0.25	−0.05	−0.30	−0.25	0.26	−0.20	−0.15	−0.20	−0.43 **	−0.41 **	−0.16	−0.05
Externalising Problems	−0.27	−0.19	0.54	−0.03	0.11	−0.38	−0.17	−0.01	−0.47	−0.24	−0.37	−0.21	0.53 **	−0.33	−0.41 **	−0.27	−0.33	−0.14	−0.38 **	−0.03
Syndrome Scales																				
Anxious/Depressed ^a^	−0.43	−0.28	0.24	−0.51	0.09	−0.56	−0.48	0.52	−0.03	−0.03	−0.16	−0.09	0.07	−0.15	0.04	−0.04	−0.34	−0.35	−0.07	−0.04
Withdrawn/Depressed ^a^	−0.10	0.20	−0.07	−0.34	0.05	0.14	−0.15	−0.00	−0.34	−0.00	−0.05	0.03	0.14	−0.11	−0.31	−0.21	−0.24	−0.24	−0.26	0.17
Somatic Complaints ^a^	0.37	0.23	0.31	−0.46	0.04	−0.28	−0.24	0.06	−0.35	−0.18	−0.20	−0.20	0.07	0.02	0.14	0.14	−0.32	−0.21	0.06	−0.16
Attention Problems ^a^	−0.35	0.02	0.37	0.17	0.16	−0.10	0.00	−0.24	−0.56	−0.22	0.01	−0.05	0.23	1.05	−0.18	−0.08	−0.05	0.00	−0.12	0.15
Aggressive Behaviour ^a^	−0.28	−0.25	0.57	−0.16	0.02	−0.47	−0.20	0.08	−0.47	−0.22	−0.18	−0.23	0.32	−0.22	−0.35	−0.00	−0.15	−0.31	−0.33	−0.01
DSM-Orientated Scales																				
Affective Problems ^a^	−0.25	0.09	0.39	−0.38	0.23	−0.25	−0.47	0.14	−0.48	−0.14	−0.40 **	−0.25	0.40 **	−0.26	−0.22	−0.23	−0.40 **	−0.32	−0.34	−0.01
Anxiety Problems	−0.26	−0.17	0.11	−0.25	0.08	−0.40	−0.18	0.38	−0.13	−0.12	−0.21	−0.19	0.12	−0.10	0.11	−0.04	−0.38	−0.38	0.04	−0.10
ADH Problems ^a^	−0.15	−0.20	0.19	0.20	0.18	−0.01	−0.13	0.06	−0.52	0.07	0.17	−0.04	0.17	−0.11	−0.31	−0.14	−0.01	−0.02	−0.16	0.10
OD Problems ^a^	−0.06	−0.38	0.56	−0.27	−0.17	−0.56	−0.25	0.19	−0.17	−0.04	−0.28	−0.20	0.39 **	0.10	−0.41 **	−0.05	−21	−0.21	−0.46 **	−0.06

Note. Coh = Cohesion; Exp = Expressiveness; Conf = Conflict; Ind = Independence; AO = Achievement Orientation; ICO = Intellectual Cultural Orientation; ARO = Active Recreational Orientation; MRE = Moral Religious Emphasis; Org = Organisation; Cont = Control. ADH Problems = Attention-Deficit Hyperactivity Problems. OD Problems = Oppositional Defiant Problems. ^a^ Scores represent Spearman’s Rho correlation coefficient. ** *p* ≤ 0.01. Bold typeface indicates significant correlation coefficient.

**Table 5:** FES descriptive data for WS and TD.

FES Subscales	Group						
	WS (*n* = 16)		TD (*n* = 46)		Between-Group Comparisons (WS-TD)		
	M (SD)	Range	M (SD)	Range	*T (59)*	*p*	Cohen’s *d*
Cohesion	54.44 (13.78)	11–**65**	56.76 (10.53)	25–**65**	−0.70	0.49	−0.20
Expressiveness	54.69 (11.22)	34–**71**	55.38 (12.06)	22–**71**	−0.20	0.84	−0.06
Conflict	44.63 (9.72)	33–**65**	47.16 (10.65)	33–**70**	−0.83	0.41	−0.24
Independence	39.00 (9.47)	29–53	41.31 (10.79)	21–**61**	−0.76	0.45	−0.22
Achievement Orientation	41.50 (11.76)	13–59	40.00 (12.67)	13–59	.41	0.68	0.12
Intellectual Cultural Orientation	45.44 (10.80)	30–**69**	55.16 (9.73)	41–**69**	−0.33 **	<0.01	−0.96 **
Active Recreational Orientation	48.44 (12.20)	23–**64**	54.47 (11.56)	33–**69**	−1.72	0.08	−0.51
Moral Religious Emphasis	51.13 (13.22)	32–**71**	45.02 (11.88)	32–**71**	1.71	0.09	0.50
Organisation	52.50 (11.03)	32–**69**	51.76 (12.30)	26–**69**	.21	0.83	0.06
Control	51.00 (11.67)	27–**70**	54.13 (11.08)	27–**76**	−0.96	0.34	−0.28

Note. Bold font indicates elevated *T* scores (≥60). ** *p* < 0.01.

## Data Availability

The data presented in this study are available on request from the corresponding author. The data are not publicly available due to privacy and ethical restrictions.

## Appendix A. Associations with SES and Parent Education

**Table A1:** Correlations between SES and parent education and CBCL and FES subscales at Time 1.

Measure	WS				TD			
	SES	Mother	Father	Parent	SES	Mother	Father	Parent
CBCL Subscales								
Summary								
Total Problems	−0.13	−0.05	−0.03	−0.05	−0.09	0.09	0.13	0.14
Internalising Problems	−0.03	0.08	0.32	0.24	0.03	0.08	0.13	0.13
Externalising Problems	−0.03	−0.07	0.05	−0.01	−0.11	0.00	0.15	0.11
Syndrome								
Anxious/Depressed	0.07	0.22	0.28	0.30	0.06	−0.11	−0.03	−0.08
Withdrawn/Depressed ^a^	0.49	0.18	0.15	0.18	0.16	−0.03	0.18	0.11
Somatic Complaints ^a^	−0.47	−0.36	−0.25	−0.25	−0.14	0.08	0.09	0.14
Attention Problems ^a^	0.17	−0.23	−0.07	−0.17	−0.05	−0.29	0.08	−0.12
Aggressive Behaviour ^a^	−0.06	−0.08	0.06	0.03	−0.11	−0.01	0.07	0.05
DSM-Orientated								
Affective Problems ^a^	−0.17	0.01	0.24	0.16	−0.20	−0.02	−0.02	−0.03
Anxiety Problems	−0.16	0.13	0.14	0.16	−0.02	−0.06	−0.11	−0.11
ADH Problems ^a^	0.01	−0.33	0.07	−0.12	0.01	−0.08	0.21	0.07
OD Problems ^a^	−0.16	−0.21	0.01	−0.08	−0.13	0.09	0.17	0.16
FES Subscales								
Cohesion	−0.16	0.25	0.38	0.38	−0.19	0.31	0.27	0.38
Expressiveness	−0.34	−0.02	0.27	0.16	−0.27	0.27	0.27	0.35
Conflict	0.14	−0.03	−0.15	−0.11	0.29	−0.22	0.10	−0.07
Independence	0.23	0.21	0.03	0.13	0.04	0.43 **	−0.20	0.12
Achievement Orientation	−0.38	−0.15	0.04	−0.06	−0.29	0.16	−0.31	−0.12
Intellectual Cultural Orientation	−0.02	0.37	0.48	0.51	0.06	0.51 **	0.23	0.47 **
Active Recreational Orientation	−0.10	0.24	0.15	0.23	−0.21	0.26	0.29	0.35
Moral Religious Emphasis	−0.05	0.04	0.38	0.26	0.07	0.34	0.40 **	0.48 **
Organisation	0.16	−0.06	0.50	0.28	0.20	0.10	0.18	0.19
Control	0.13	0.00	0.49	0.30	0.02	−0.07	−0.15	−0.14

Note. SES = Socioeconomic Status at Time 1. Mother = Mother total years of education. Father = Father total years of education. Parent = Average parent total years of education. ADH = Attention-Deficit Hyperactivity. OD = Oppositional Defiant. ^a^ Scores represent Spearman’s Rho correlation coefficient. ** *p* < 0.01.

**Table A2:** Correlations between SES and parent education and CBCL at Time 2.

CBCL Subscales	WS				TD			
	SES2	Mother	Father	Parent	SES2	Mother	Father	Parent
Summary								
Total Problems	−0.49	−0.31	−0.35	−0.39	0.23	−0.22	0.11	−0.06
Internalising Problems	−0.45	−0.35	−0.15	−0.29	0.28	−0.28	−0.18	−0.29
Externalising Problems	−0.38	−0.23	−0.49	−0.44	0.22	−0.14	0.25	0.08
Syndrome								
Anxious/Depressed ^a^	−0.41	0.45	−0.28	−0.48	0.28	−0.15	−0.14	−0.17
Withdrawn/Depressed ^a^	−0.00	0.11	0.05	0.07	0.03	−0.28	0.02	−0.13
Somatic Complaints ^a^	−0.29	−0.40	−0.16	−0.36	0.06	−0.24	−0.25	−0.28
Attention Problems ^a^	−0.03	0.00	−0.16	−0.09	0.16	0.05	0.13	0.13
Aggressive Behaviour ^a^	−0.37	−0.21	−0.48	−0.38	0.41 **	−0.12	0.14	0.05
DSM-Orientated								
Affective Problems ^a^	−0.36	−0.19	−0.08	−0.17	0.19	−0.32	0.15	−0.08
Anxiety Problems	−0.58	−0.31	−0.30	−0.36	0.17	−0.03	−0.16	−0.13
ADH Problems ^a^	−0.13	0.00	−0.13	−0.02	0.27	0.03	0.19	0.12
OD Problems ^a^	−0.26	−0.25	−0.34	−0.32	0.32	−0.15	0.17	0.04

Note. SES = Socioeconomic Status at Time 2. Mother = Mother total years of education. Father = Father total years of education. Parent = Average parent total years of education. ADH = Attention-Deficit Hyperactivity. OD = Oppositional Defiant. ^a^ Scores represent Spearman’s Rho correlation coefficient. ** *p* < 0.01.

## Appendix B. Associations with Demographic Variables

**Table A3:** Correlations between demographic variables and CBCL at Time 1 and 2 in WS and TD.

CBCL Subscales	WS					TD				
	Sex	Age	Global DQ	Verbal DQ	NV DQ	Sex	Age	Global DQ	Verbal DQ	NV DQ
Time 1										
Summary										
Total Problems	−0.39	0.27	0.17	−0.18	0.29	−0.04	−0.09	−0.13	−0.14	−0.08
Internalising Problems	−0.09	−0.08	0.32	−0.14	0.08	0.07	0.25	−0.07	−0.05	−0.08
Externalising Problems	−0.20	0.32	0.34	0.05	0.32	−0.11	−0.13	−0.17	−0.23	−0.12
Syndrome										
Anxious/Depressed	−0.16	0.05	0.37	0.03	0.27	0.21	0.40 **	−0.34	−0.01	−0.01
Withdrawn/Depressed ^a^	0.47	−0.51	0.04	0.03	−0.58	−0.04	0.07	−0.00	0.06	−0.04
Somatic Complaints ^a^	−0.25	0.27	0.03	−0.02	0.31	−0.13	0.01	0.03	−0.08	0.03
Attention Problems ^a^	−0.03	0.29	−0.04	−0.01	−0.01	−0.04	−0.07	−0.09	−0.20	−0.10
Aggressive Behaviour ^a^	−0.32	0.25	0.12	0.03	0.25	−0.11	−0.09	−0.13	−0.22	−0.13
DSM-Orientated										
Affective Problems ^a^	−0.45	0.16	0.36	0.24	0.22	0.04	−0.35	−0.10	−0.06	−0.06
Anxiety Problems	−0.17	0.37	0.49	0.19	0.43	0.27	0.37	−0.06	−0.06	−0.06
ADH Problems ^a^	−0.04	0.19	−0.14	−0.18	0.03	−0.03	−0.36	−0.13	−0.19	−0.09
OD Problems ^a^	−0.18	0.35	0.20	0.16	0.27	−0.04	−0.03	−0.16	−0.16	−0.19
Time 2										
Summary										
Total Problems	−0.19	0.40	0.33	0.05	0.14	0.01	0.12	−0.09	0.07	−0.10
Internalising Problems	−0.24	−0.04	0.05	−0.35	−0.23	0.29	0.31	−0.08	0.06	−0.06
Externalising Problems	−0.19	0.45	0.37	0.09	0.26	−0.04	0.02	−0.02	0.14	−0.05
Syndrome										
Anxious/Depressed ^a^	−0.38	0.19	0.09	−0.15	0.12	0.39 **	0.33	−0.13	−0.02	−0.06
Withdrawn/Depressed ^a^	−0.10	−0.53	−0.15	−0.15	−0.55	0.03	0.01	0.10	0.06	0.22
Somatic Complaints ^a^	−0.14	0.12	0.13	−0.03	−0.05	0.12	0.10	−0.14	−0.03	−0.13
Attention Problems ^a^	0.28	0.07	0.40	0.40	−0.10	−0.14	0.17	−0.07	−0.07	−0.12
Aggressive Behaviour ^a^	−0.26	0.44	0.37	0.24	0.26	−0.05	0.16	0.01	0.05	0.03
DSM-Orientated										
Affective Problems ^a^	−0.26	0.05	0.16	0.08	−0.21	0.17	−0.16	−0.08	0.08	−0.03
Anxiety Problems	−0.20	0.38	0.25	−0.05	0.22	0.34	0.24	−0.14	0.02	−0.14
ADH Problems ^a^	0.36	0.14	0.35	0.40	0.18	−0.18	0.15	−0.09	−0.14	−0.08
OD Problems ^a^	−0.26	0.51	0.28	0.19	0.29	−0.03	0.04	−0.04	0.14	−0.07

Note. Age = Chronological Age. DQ = Developmental Quotient. NV = Nonverbal. ADH = Attention-Deficit Hyperactivity. OD = Oppositional Defiant. ^a^ Scores represent Spearman’s Rho correlation coefficient. ** *p* < 0.01.
